# Natural and Vaccine-Induced Immunity in the Post-Pandemic Era: Convergence, Divergence, and Unfinished Challenges

**DOI:** 10.3390/vaccines13121251

**Published:** 2025-12-17

**Authors:** Chloe Dimeglio

**Affiliations:** Toulouse Institute for Infectious and Inflammatory Diseases (INFINITY), Institut National de la Santé et de la Recherche Médicale (INSERM), Unité Mite de Recherche 1291, Centre National de la Recherche Scientifique (CNRS), Unité Mite de Recherche 5051, 31300 Toulouse, France; dimeglio.c@chu-toulouse.fr

The global response to SARS-CoV-2 has advanced at unprecedented speed: vaccines developed in record time, mass immunization campaigns, and real-world data confirming reductions in severe disease and death [1,2]. Yet, as we mark a shift into a more endemic phase of the pandemic, the central question is no longer whether we can provide immunity, but rather how well, how durably, and how equitably we can deliver it. The Special Issue of *Vaccines* titled “Influence of Natural and/or Vaccine Immunity on the Dynamics of SARS-CoV-2” was launched precisely to interrogate this complexity—how natural infection and vaccination, alone or in combination, shape immunity in the face of evolving viral dynamics.

This collection offers crucial insights into three interlinked domains: the heterogeneity of immune acquisition and waning, the operational significance of hybrid immunity, and the threat posed by viral evolution in undermining protection. While the fundamental tenets—vaccination is key [3], immunity wanes [4], variants matter [5]—are well established, the contributions to this Special Issue refine our understanding and signal new strategic imperatives.

Several contributions emphasize that the quality of immune memory varies markedly with the variant first encountered. Oluka et al. [6] followed individuals in Uganda initially exposed to A.23.1 and revealed durable yet uneven cross-neutralization against other strains over 427 days. This provides rare evidence that immune imprinting—beneficial or limiting—depends on the immunological “first impression”. Hybrid immunity remains a critical asset among those at highest occupational risk. In healthcare workers, Chivu et al. [7] reported lower reinfection rates during Omicron dominance among individuals with mixed infection and vaccination history, emphasizing the sustaining role of repeated antigenic stimulation. These findings not only align with established global observations but also extend them by variant-specific granularity—a cornerstone for future vaccine guidance. Community-scale evidence from Gim et al. [8] clarifies that vaccination reduces both symptomatic reinfections and the speed of recurrence, confirming its population benefit despite immune evasion by Omicron sublineages. Yet heterogeneity remains a decisive factor. Through immune profiling after three vaccine doses, Ko et al. [9] identified age, compromised immunity, and specific comorbidities as critical predictors of impaired humoral and cellular responses—reinforcing the need for stratified booster policies rather than universal schedules. The study by Meyers et al. [10] further challenges assumptions: Vitamin D levels—long suspected to impact vaccine response—showed no significant correlation with antibody generation in nursing home residents and staff, suggesting that routine supplementation is not a proxy for immune optimization in vaccination. Dimeglio et al. [11] provide one of the most actionable contributions: quantitative antibody thresholds required to protect against Omicron BA.1/BA.2. Although higher than for previous variants, such correlates remain essential tools for public health decision-making, booster deployment, and clinical risk stratification. Sero-surveillance among children in Saudi Arabia by Asseri and Alsabaani [12] underlines that immunity levels were substantial in the pre-vaccination era, revealing widespread silent transmission and the need for child-focused monitoring strategies. Chang-Rabley et al. [13] synthesize evidence on individuals with inborn errors of immunity and call for bespoke vaccination strategies, including monoclonal antibodies and adjuvant-enhanced formulations—a still-underfunded horizon in global immunization equity. Padariya & Kalathiya [14] advance the future of vaccine engineering through ferritin nanocage platforms displaying variant RBDs—a versatile tool for pan-variant vaccine development. Meanwhile, Buchhorn [15] sheds light on dysautonomia in children following infection and, more rarely, vaccination—a reminder that post-acute sequelae remain a critical frontier requiring multidisciplinary research rather than dismissal. Finally, Salvagno & Lippi [16] present a pragmatic contribution: spike IgG measurements strongly correlate with total antibody responses, streamlining sero-monitoring protocols for large cohorts. Chavda et al. [17] offer a timely perspective on mRNA vaccine technology, reminding the reader that despite recent deployment, the platform is founded on decades of immunobiology research—a narrative crucial to counter vaccine skepticism.

Crucially, this Special Issue adds value over the extant evidence base in three ways. It expands the focus on under-served populations (immunocompromised and hybrid immunity cohorts), it underscores improvements in methodology (linking immunologic and epidemiologic data; variant-specific neutralization), and it advances an operational lens: how immune trajectories can inform policy (boosting schedules, variant-adapted vaccines, and equity of access). While the previous literature laid the groundwork for waning immunity and variant impact [18] on durability of natural and vaccine-mediated immunity), this collection elevates it into a more actionable domain.

On the basis of these insights, this Special Issue offers the following strategic recommendations:

## 1. Refined Immunological Surveillance

Surveillance systems should measure both humoral (neutralizing antibodies; binding IgG) and cellular immunity (T-cell responses; memory B cells), particularly in populations with increased vulnerability. Evidence demonstrating variability in neutralization breadth and durability across exposure histories supports the need for immune history stratification [6,7,9]. Moreover, variant-specific immune escape requires the integration of updated neutralization assays into public health practice [6,11].

## 2. Tailored Vaccination and Booster Strategy

Booster policies must incorporate prior infection profiles: hybrid immunity confers enhanced resistance against reinfection in frontline workers [7], while vaccination alone remains protective against recurrent disease at the community level [8]. Individuals with underlying immune impairment exhibit suboptimal vaccine-induced responses [9,13], justifying enhanced regimens and frequent monitoring. Vaccine antigen updates must reflect the dynamic variant landscape to avoid mismatches in immune recognition [14,17].

## 3. Dynamic Modeling and Planning

Epidemiological models should integrate waning immune markers, variant evolution, and repeated antigen exposure, as supported by longitudinal evidence showing altering neutralization profiles over time [6] and changes in reinfection risk during Omicron waves [7,8]. Incorporating such parameters will better guide booster deployment and resource prioritization.

## 4. Focused Research on Immune Correlates and Special Populations

The establishment of quantitative correlates of protection remains essential [11]. Long-term follow-up is needed across age and exposure groups, including children—who demonstrated substantial pre-vaccination seropositivity [12]—and individuals with inborn errors of immunity, who may benefit from tailored immunization strategies and alternative biologics [8]. Emerging observations regarding post-acute sequelae, including dysautonomia in pediatric cases after infection and/or vaccination, highlight an ongoing need for protective strategies that minimize both short- and long-term harm [15].

## 5. Equity in Access and Implementation

Global control of SARS-CoV-2 requires the prevention of immunity gaps between well-resourced and underserved populations. Hybrid immunity must not become a privilege tied to inequitable infection and vaccine access [7,8]. Efforts to expand variant-adapted vaccine availability, reinforce genomic and immunologic surveillance, and sustain public communication are imperative to ensure that vaccination remains a universal benefit rather than a selective advantage [10,14,17].

In conclusion, the body of work compiled in this Special Issue confirms that immunity to SARS-CoV-2 is not merely a milestone but a journey—one characterized by complexity, variation, and evolution. The scientific community has moved from demonstrating what is possible to grappling with what is optimal. The ambition now must be to translate this rich evidence into resilient, adaptive, and equitable immunization systems: systems that do not just cover populations but protect them durably across viral variants, immunological histories, and global settings.

The path ahead remains challenging—but the tools, the data, and the strategic clarity are now in hand. Our collective responsibility is to deploy them effectively so that immunity against SARS-CoV-2 becomes not a variable state but a sustained reality.
